# An evaluation of an Extension for Community Healthcare Outcomes (ECHO) intervention in cancer prevention and survivorship care

**DOI:** 10.1186/s12911-022-01874-x

**Published:** 2022-05-17

**Authors:** Zheng Z. Milgrom, Tyler S. Severance, Caitlin M. Scanlon, Anyé T. Carson, Andrea D. Janota, Terry A. Vik, Joan M. Duwve, Brian E. Dixon, Eneida A. Mendonca

**Affiliations:** 1 Center for Biomedical Informatics, Regenstrief Institute, 1101 West Tenth Street, Indianapolis, IN 46202 USA; 2 Richard M. Fairbanks School of Public Health, Indiana University, 1050 Wishard Boulevard, Indianapolis, IN 46202 USA; 3 Riley Hospital for Children, 705 Riley Hospital Drive, Indianapolis, IN 46202 USA; 4 Indiana University School of Medicine, 340 West Tenth Street, Fairbanks Hall, Suite 6200, Indianapolis, IN 46202 USA; 5 Kansas Department of Health and Environment, Curtis State Office Building, 1000 SW Jackson St, Topeka, KS 66612 USA

**Keywords:** Telemedicine, Education, Continuing, Population health, Cancer control, Evaluation

## Abstract

To improve cancer care in Indiana, a telementoring program using the Extension for Community Healthcare Outcomes (ECHO) model was introduced in September 2019 to promote best-practice cancer prevention, screening, and survivorship care by primary care providers (PCPs). The aim of this study was to evaluate the program’s educational outcomes in its pilot year, using Moore’s Evaluation Framework for Continuing Medical Education and focusing on the program’s impact on participants’ knowledge, confidence, and professional practice. We collected data in 22 semi-structured interviews (13 PCPs and 9 non-PCPs) and 30 anonymous one-time surveys (14 PCPs and 16 non-PCPs) from the program participants (hub and spoke site members), as well as from members of the target audience who did not participate. In the first year, average attendance at each session was 2.5 PCPs and 12 non-PCP professionals. In spite of a relatively low PCP participation, the program received very positive satisfaction scores, and participants reported improvements in knowledge, confidence, and practice. Both program participants and target audience respondents particularly valued three features of the program: its conversational format, the real-life experiences gained, and the support received from a professional interdisciplinary community. PCPs reported preferring case discussions over didactics*.* Our results suggest that the Cancer ECHO program has benefits over other PCP-targetted cancer control interventions and could be an effective educational means of improving cancer control capacity among PCPs and others. Further study is warranted to explain the discrepancies among study participants’ perceptions of the program’s strengths and the relatively low PCP participation before undertaking a full-scale effectiveness study.

**Supplementary Information:**

The online version contains supplementary material available at 10.1186/s12911-022-01874-x.

## Introduction

It is estimated that two in five Indiana residents will be diagnosed with cancer in their lifetimes [1–3]. With ongoing efforts to provide community resources and expand care coverage for cancer screening and prevention, especially for tobacco cessation as well as breast and cervical cancers, cancer incidence has been gradually decreasing in the state. However, cancer is still the second leading cause of death in Indiana [3], and the state’s population of cancer survivors continues to grow from a total of 298,425 in 2015 [3]. Compared to the rest of the United States, the state’s age-adjusted mortality rate was 17% higher in 2017 [4]. At the same time, the costs of cancer care are becoming more unaffordable [5, 6]. The national annual cancer-related health care cost was approximately $183 billion in 2015 and was estimated to grow to $222.2 billion in 2025 [5]. Controlling these costs while taking steps to both prevent cancer and take care of cancer patients and survivors require a collective effort [1]. Previous studies found that a physician’s recommendation was the primary factor influencing whether a patient was screened for cancer [7–9]. However, primary care providers (PCPs), including physicians and advanced practitioners, face challenges in providing primary prevention of cancer. Barriers identified in prior studies include lacking adequate resources to keep up-to-date with emerging evidence and changing community resources as well as experiencing high burnout rates [10, 11]. To address these challenges, the Indiana Cancer Consortium established as priorities educating Indiana’s healthcare workforce to implement evidence-based strategies and convening multi-sector discussions of cancer-related challenges [1].

In September 2019, the Indiana Cancer Consortium collaborated with the Indiana University (IU) Richard M. Fairbanks School of Public Health and the Indiana University Purdue University at Indianapolis (IUPUI) ECHO Center to launch the Cancer Screening, Prevention, and Survivorship ECHO program. Extension for Community Healthcare Outcomes (ECHO©) programs are becoming an important resource for capacity-building in the health sector; to date, 52 institutions globally and 34 institutions in the United States have adopted the ECHO model to provide remote cancer-related education [12]. The ECHO model is a telementoring intervention design that, in a wheel-like configuration, connects frontline healthcare workers at various locations (“spokes”) with experts at an academic center (“hub”) at regularly scheduled times. In the Cancer ECHO program, the hub experts facilitate and guide the frontline participants through two capacity-building components in each session: hub-led didactics on curriculum topics and spoke-led discussions of de-identified patient cases. Over a decade ago, the first ECHO program, which focused on the care of patients with Hepatitis C, successfully demonstrated that Hepatitis C care delivered by ECHO-trained rural physicians was equally effective as that given at the University of New Mexico [13]. Additional evidence of the feasibility of the ECHO model as a cost-effective intervention addressing specific cancers in the United States and general cancer-related topics overseas has been reported [14–17]. To educate PCPs across Indiana and connect them with resources in the Indiana Cancer Consortium and Fairbanks School of Public Health as well as their partnerships, the multi-point teleconferencing technology in the ECHO model makes it possible to put collaborative support within the reach of PCPs and involve them in continuous statewide conversations on cancer-related challenges.

In the first year of the Cancer ECHO program (from September 17, 2019, to September 16, 2020), 22 sessions were held. The aim of this study was to evaluate the program’s educational outcomes in its pilot year, using Moore’s Evaluation Framework for Continuing Medical Education and focusing on the program’s impact on participants’ knowledge, confidence, and professional practice. Assessing input from the program’s participants and target audience will help us begin to identify the mechanisms that contribute to and limit the effectiveness of the ECHO model for providing continuing education to health care professionals regarding cancer care and screening.

## Methods

This exploratory study used a mixed-methods approach (interviews and a survey). To evaluate the Cancer ECHO program’s learning outcomes, we used Moore’s Evaluation Framework for Continuing Medical Education [18]. Moore’s framework is a seven-level pyramid model used to assess the outcomes of continuing education activities for clinicians. In our study, we assessed the program using the first five levels of Moore’s framework: participation (Level 1), satisfaction (Level 2), impact on knowledge (Level 3), impact on confidence (Level 4), and impact on professional practice (Level 5). We also measured the program’s impact on professional burnout with two single-item measures on burnout (emotional exhaustion and sensitivity to patients’ feelings) adapted from the full Maslach Burnout Inventory (MBI) [19–21].

This study was approved by the IU Institutional Review Board. We analyzed administrative data from the IUPUI ECHO Center to determine program participation (Level 1). To assess Levels 2–5, we conducted semi-structured interviews and an anonymous one-time survey with questions developed from Moore’s framework and the two MBI items.

The pilot year of the program coincided with the first year of the SARS-CoV-2 pandemic, and only two learners achieved a completion certification at its end (based on the requirement for attending at least 50% of the scheduled sessions). Considering this challenge, we adopted a wide recruitment strategy for our study to maximize the number of participants. We recruited from three distinct groups: (1) the learners at spoke sites who attended at least one complete session during the year (also called the spokes participants); (2) members of the expert panel and IUPUI ECHO leadership (also called the hub participants); and (3) individuals in the program’s target audience who were aware of the Cancer ECHO program but did not participate in a session (also called potential spokes [PS] participants). Recruiting from groups that participated in the program and peers who did not allowed us to compare those groups’ perspectives and thus explore the mechanisms that may contribute to or limit the program’s effectiveness. We utilized the mailing list of learners in actual program sessions to recruit the spokes participants. The PS participants were recruited from mailing lists of registrants for the Cancer ECHO program (since some registered but did not attend) and participants in IU PCP-targetted ECHO programs on other topics. Additionally, the program attracted learners from many disciplines other than PCPs, and they constituted an important part of the multidisciplinary dialogs in the program [22]. Considering this fact and our goal of maximizing study participation, we recruited both PCPs and non-PCPs from the PS group although we attempted to recruit as many PCPs as possible since they were the original target audience for the Cancer ECHO program. This mixed-group approach also allowed us to triangulate the quantitative and qualitative data based on different roles.

Study participants were invited to take the online survey or participate in an interview or do both. The survey began with a question about the respondents’ relationship to the Cancer ECHO program (participated, heard about but did not participate, etc.) and a few demographic and professional questions. Items then asked respondents to rate their satisfaction with the program; rate its impact on their knowledge, confidence, and professional practice; and, using a Likert scale, rate their emotional exhaustion and insensitivity to patients before and after participating in the Cancer ECHO program. (For survey questions, see Additional file 1: Survey Questions). The interviews were guided by a semi-structured interview protocol, in which questions varied slightly based on whether the interviewee was from the spoke, hub, or PS group. Questions addressed interviewees’ role in the program, motivation for attending and satisfaction with the program, and how the program affected (or could affect) participants’ knowledge, motivation, and professional practice. The final questions asked interviewees to compare this program with other continuing medical education, identify barriers to attending, and suggest improvements. (For the full interview guide, see Milgrom et al. [23]).

Data were collected between May 2020 and September 2020. Incentives of $10–40 were provided to study participants to promote recruitment during the pandemic. The survey was administered through Qualtrics and analyzed on SPSS. The one-on-one interviews took place via the internet and telephone. A single independent researcher (Z.M.) conducted all the interviews. NVivo machine-transcription service was used to transcribe the recorded interviews and pair them with manual audits for accuracy. Using NVivo 12, we first coded the semi-structured interviews deductively with Moore’s framework. Then we analyzed the new themes that arose inductively from the data but were not covered by Moore’s framework. An iterative, code-recode, and group consensus approach was employed for the theme development. Two research team members (Z.M. and E.M.) independent of the program operation met regularly to develop an initial set of codes, then discuss and organize the emerging themes iteratively until a code consistency was achieved. Following that, the full team met and reached a consensus on the final themes; then, findings across groups were compared and summarized within and across the interviews and surveys. The recommendations to the Cancer ECHO program were generated from the study findings.

## Results

### Program settings and participation

#### Moore’s level 1: program participation

The 22 sessions were typically 1.5 h in length and consisted of a 20-min didactic presentation and a one-hour case discussion. The 22 didactic presentations by the hub team covered a range of topics in screening, prevention, and survivorship. All but one of the 17 case discussions were about cancer survivorship. A total of 147 unique individuals attended the Cancer ECHO at least once during this period. Of them, 16 (10.8%) were PCPs (physicians and advanced practice providers). An average of 2.5 PCPs (17.2%) and 12 other professionals attended each session, and 90.9% of the sessions had at least one PCP spoke participant. The most common non-PCP attendees were cancer educators, navigators, public health workers, or administrators.

#### Study participation

We interviewed 15 program participants (12 spokes learners and three hub members) and seven PS individuals. Among these 22 interviewees, five spoke and six PS learners were PCPs. Seven (58.3%) spokes and seven (100%) PS interviewees had experience attending other ECHO programs. Sixteen of the 22 interviewees were females. Participation data showed that 90.9% of the Cancer ECHO sessions had at least one of the 12 spoke interviewees in them, and 63.6% of the sessions had at least one of the five spoke PCP interviewees. Survey respondents were 14 spoke and 16 PS learners. Among these 30 survey respondents, four spoke and ten PS learners were PCPs; 24 were females. Regarding the setting of their practice, 14 (46.7%) were urban, and 11 (36.7%) were suburban or rural while the rest chose “Others” or did not answer. Nine of the survey respondents (six spoke and three PS learners) also participated in interviews..) The interviewees quoted below are identified by role—doctor of medicine (MD), doctor of dental surgery (DDS), and family nurse practitioner (FNP)—and their assigned number in their participant group (S, H, or PS).

### Moore’s level 2: satisfaction

**Fig. 1:**
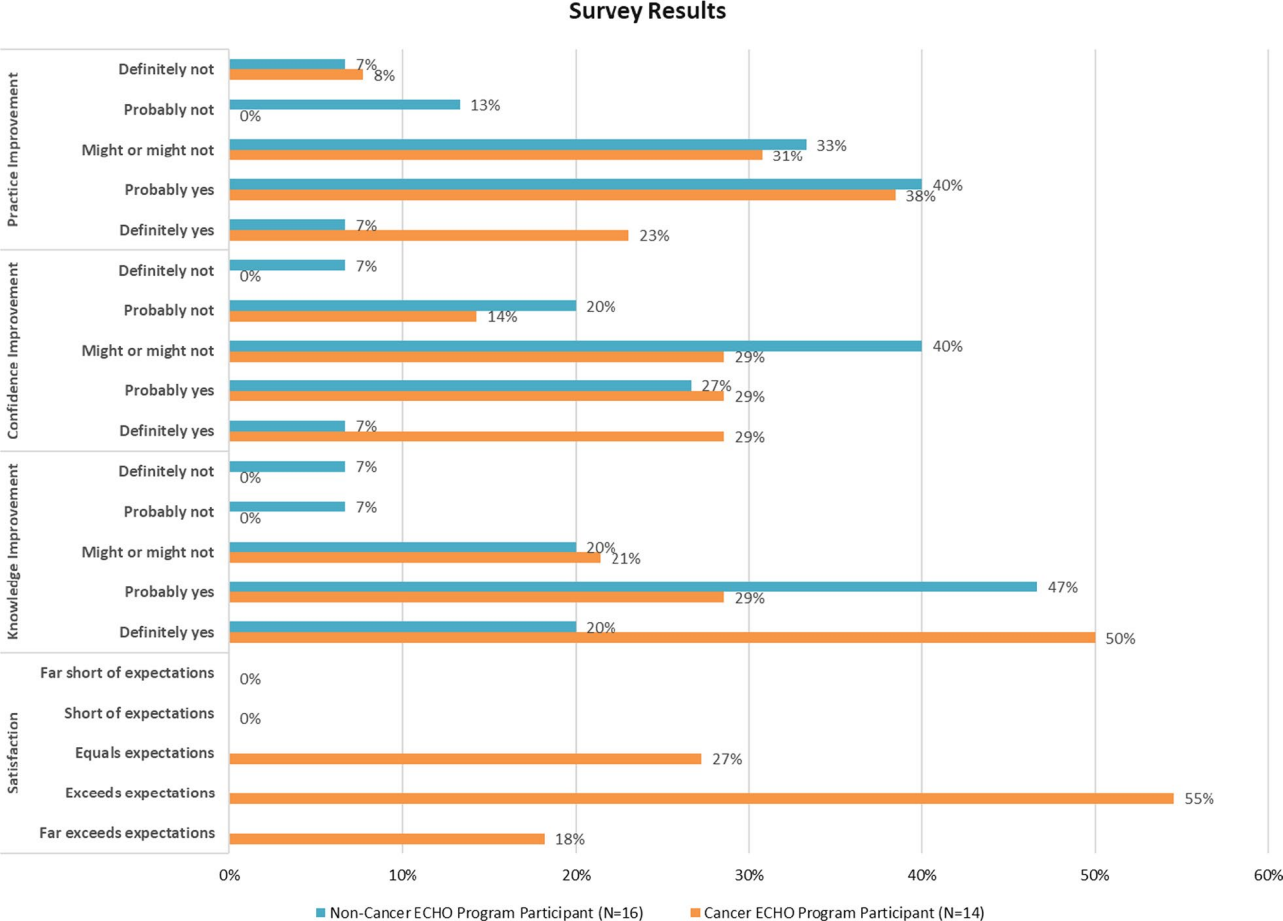
Survey results on self-reported satisfaction and knowledge, confidence, and practice improvement

Fourteen spokes learners rated their satisfaction with the program in the survey on a five-point Likert scale. The program met 100% of and exceeded 73% of both PCPs’ and non-PCPs’ expectations (see Fig. 1).

Overall, the interviewees (12 spokes learners [S] and three hub members [H]) reported being satisfied with the program. When we asked why they liked the program, six (four PCPs) interviewees mentioned the conversational format; three (two PCPs) said connecting to the multidisciplinary community; and five (three PCPs) said the real-world experiences they gained. Regarding aspects of the program that did not meet their expectations, S4 [MD] said, “it just didn’t seem as engaging.” Hub members also shared their perceived challenges, with H2 [MD] saying, “We’re having a tough time getting spoke sites to find good cases to present.”

### Moore’s levels 3 to 5: effects on knowledge, confidence, and practice

On the survey, 14 spokes and 16 PS learners self-rated their knowledge, confidence, and practice changes from any source during the period of the first year of the Cancer ECHO program (see Fig. 1). On the five-point scale, the spokes learners gave higher ratings to their improvement in all three categories than the PS learners: 79% versus 67%, 58% versus 34%, and 58% versus 47% of the spokes and PS learners respectively answered “definitely yes” or “probably yes” to their improvement in knowledge, confidence, and practice. Among them, 50% versus 20%, 29% versus 7%, and 23% versus 7% of the spokes and PS learners respectively answered “definitely yes.”

In the interviewees with spokes learners, S11 [MD] said, “knowledge change, that is tremendous.” S12 [MD] said, “I can say that my knowledge, confidence has increased, has increased dramatically.” They reported that their practice benefited from the program by having more options and developing a deeper empathy for what their patients were going through. S6 [FNP] shared how her practice changed through presenting a case discussion: “I think it’s refreshing to hear a different perspective. So, in the case I actually presented…it was very challenging, and I was prepared to just say, OK, we’ve done all we can with this person, and now that the ball’s in their court. But after talking with several people who participated [in the Cancer ECHO] that week, they really empathized and really brought forward potentially other barriers and other factors that I had not really considered. So, … when I left that presentation, [it] made me think, OK, I need to be more gracious and more patient, and stick with this case instead of at this point just kind of being done. So that was helpful.”

Three themes emerged from the interviews that described the strengths of the Cancer ECHO program, which were consistent with the three themes in the interviewees’ answers for satisfaction (see Table 1). First, the spokes participants said they benefited from the conversational and interactive format. “It's been good to hear the [didactics] presentations, which is what I initially thought was gonna be the most helpful. But actually, the case presentation and discussion component where you have a question and answer has been surprisingly more beneficial, I think, at times and help create change to my practice,” said S6 [FNP]. PS6 [DDS] agreed, saying, “it just seemed really interactive.” Second, interviewees commented that learning is best when the content is real-world experiences. For this reason, many interviewees, especially PCPs, greatly favored the case discussion over the didactic component. S5 [MD] reported, “I probably like the case discussions the best, honestly, just because I think when you start with the case, it triggers a memory in my mind as a patient who is like that or something. Whereas if it's just a didactic session, it's helpful and nice, but it's harder to put into context. It's easier for me to lose interest.” “I like the idea of having a case presented, which is great,” said PS7 [MD]. Lastly, most of the participants highlighted changes they’d made based on learning from other participants, exploring more care options through collaboration, adapting the new resources and ideas into practice, and having better communications with patients. A PCP commented, “That's what I like about the ECHO, [that] they have a different perspective, and it's not just physicians. It's either a social worker, which I rarely hear in practice, and you get to understand how important they are, or your physical therapists or your rehab people. The nurse you see most of the time, but we don't really get their perspective on what's going on. That makes it more of a complete, almost team effect on the patient.” This aspect was also appreciated by the PS participants. “Everyone is invited to talk, there is no judgment,” said PS4 [MD].

**Table 1:** Emergent themes regarding the program’s strengths as expressed by the interviewees

Themes	Interviewees	
Format: Conversational and interactive (in the case discussion component)	PCPs: 12 out of 13 (4/5 spoke, 6/6 PS, 2/2 hub) Non-PCPss: 7 out of 9 (except S3 [MPH] liked didactics better, and S9 [CHES] liked didactics and case discussion equally)	Spoke: 9 out of 12 Potential Spoke: 7 out of 7 Hub: 3 out of 3
Content: Real-world experiences	PCPs: 13 out of 13 (5/5 spoke, 6/6 PS, 2/2 hub) Non-PCPs: 7 out of 9 (except S3 [MPH] liked didactics better; PS2 [LCSW] did not mention this theme)	Spoke: 10 out of 12 Potential Spoke: 6 out of 7 Hub: 3 out of 3
Participant and community: A nonjudgmental, safe learning environment and support from a multi-disciplinary community	PCPs: 13 out of 13 (5/5 spoke, 6/6 PS, 2/2 hub) Non-PCPs: 8 out of 9 (except S7 [MCHES] did not mention this theme)	Spoke: 11 out of 12 Potential Spoke: 7 out of 7 Hub: 3 out of 3

PCPs are primary care providers and specialist physicians in the spoke, potential tpoke, and hub groups

CHES, Certified Health Education Specialist; ECHO, Extension for Community Healthcare Outcomes; LCSW, Licensed Clinical Social Worker; MPH, Master of Public Health; MCHES, Master of Certified Health Education Specialist; PS, interviewee in potential spoke group; S, interviewee in spoke group

Some interviewees did not think the Cancer ECHO program had changed their practice yet, citing these reasons: (1) their participation in the program was still too limited to see changes, and they faced barriers to maintain high participation, such as time commitment; (2) some of the topics were not directly pertinent to their practices; and (3) since the learning happened from discussion with other participants, the quality of the program was related to the program participants. H3 [MD] mentioned as a concern, “I don’t think we are capturing primary care providers.” The spoke PCPs thought the didactic presentations were less effective than the case discussions, which is consistent with perspectives expressed by the PS PCPs.

### Additional measurement of the effects on burnout

We used changes in emotional exhaustion and insensitivity to evaluate the program’s impact on providers’ potential burnout. Though many PCPs did not report significant general emotional exhaustion changes, they did speak highly of the program’s potential to improve their burnout levels with features that contributed to the program’s effectiveness. On the survey, 40% and 60% respectively of participants reported the Cancer ECHO program had a positive impact on their emotional exhaustion and insensitivity levels. (See Additional file 2: Survey Results on Self-Reported Burnout Change). As H3 [MD] described it, “medicine is draining when you feel like you have nothing else to offer.” S5 [MD] thought having a safe place, such as the Cancer ECHO program, to discuss the mistakes providers have made or dealt with could help reduce emotional exhaustion. Our study participants reported feeling the program helped them to better empathize with patients and be more understanding and sensitive to what their patients were going through.

## Discussion

Grounded in Situated Learning Theory and Communities of Practice, the goal of Project ECHO is to use a telementoring model with didactics and case-based learning to train care providers and to move knowledge to where providers and patients are [24, 25]. While traditional ECHO programs, as described by Severance et al., focus on clinical topics, the Cancer Screening, Prevention, and Survivorshop ECHO program covers a broad spectrum of topics in public and population health as well as clinical care. The broad coverage aims to support the various needs of frontline PCPs across the state of Indiana to implement cancer prevention and provide care for cancer survivors. Despite a relatively low PCP participation (Moore’s Level 1) in its pilot year, the program overall received very positive satisfaction scores (Moore’s Level 2) and positive feedback from the spokes learners regarding Moore’s Levels 3 to 5. In the survey results, the program met or exceeded the expectations of 100% of both PCPs and non-PCPs (Moore’s Level 2). In the qualitative results, there were three features that all the interviewees valued in the Cancer ECHO program: the conversational format, gaining real-life experiences, and receiving support from a professional interdisciplinary community. Our results support the contexualization of Project ECHO’s grounding theories of change in cancer control. Respondents believed the case discussions in particular were, or could be, the mechanisms of both satisfaction and improvement in knowledge, confidence, and practice. These features, according to the interviewees, separated the Cancer ECHO program from other continuing education activities on cancer control. However, not having cases from spokes to present was a struggle for the program, which was also found to be a challenge in an ECHO program about tobacco cessation [16]. It is important to note the PCPs’ resistance to the didactics component in the Cancer ECHO program, which to our knowledge is a finding not previously reported in any ECHO context. Of note, the cancer-related ECHO programs in previous studies had a narrower focus or involved only one-time training outside of the United States [14–17].

Unlike the non-PCPs, some PCPs in our study reported feeling they did not have a significant knowledge gap in the didactics content on cancer control. They perceived that guidelines are limited because such guidelines do not recognize the nuances of individual health and social circumstances in the PCPs’ own practices. These opinions were consistent with the physicians’ resistance to the other guideline adherence efforts on cancer preventive medicine, such as electronic health record (EHR) alerts and automated guidelines [26]. According to the PCPs, EHR alerts, as the primary tool for guideline dissemination and awareness reinforcement, were able to identify cases not meeting the guideline recommendations at the point of care. To implement prevention guidelines, Doherty et al. found that communication means, such as clinician consensus-building working groups and meetings, beyond electronic medical records and didactics were necessary [27]. Indeed, the case discussion in the Cancer ECHO program offers a platform to learn case-specific strategies, share informal knowledge, and make collaborative decisions. These aspects set the program apart from other educational communication means, highlight its unique role in cancer control, and overcome the limitations of regular didactics and EHR alerts.

Though never having participated in the Cancer ECHO program, the PS study participants also said they believed the program had features that made it desirable in the context of cancer control. The PS learners’ perception that the program was desirable helped us understand that their lack of attendance was not because their understanding of the program’s strengths differed substantially from that of learners who did participate. Rather, their non-attendance may have been because they failed to see the program would have a positive learning impact on them. Another finding possibly related to PCPs’ motivations to attend was that the PCP-led case discussions almost always chose survivorship topics, whereas the didactic presentations also addressed prevention and screening. These findings of PCPs’ preferences for case discussion and survivorship topics emphasize the importance of understanding the PCPs’ unmet needs that may influence their participation. According to our interviewees, the extent of PCP participation also had an impact on the session quality, motivated other PCPs to join, and was related to the longitudinal program impact. A further in-depth investigation into the identified PCP participation issue is warranted before undertaking a full-scale effectiveness and scalability study. The barriers and incentives to PCPs’ participation were the focus of our prior study, which revealed desired adaptations to better meet PCPs’ needs [23].

## Considerations for future program development

In the future, we suggest consideration of two different possible directions for the Cancer ECHO program. The first direction is to change its original target of PCPs to instead target a broader range of health care professionals. In its pilot year, the program received interest from many non-PCP professionals, who were also more open than PCPs to the topics of cancer screening and prevention as well as the guideline-reviewing activities in the didactics component. The second direction is to continue to target PCPs, but introduce adaptations to increase the likelihood of their participation. Our results suggest the program could be effective in training PCPs. However, new strategies are needed to encourage their participation and to balance the PCPs’ needs and the program aims.

## Study limitations

Our study was limited, first, by the fact that we started our evaluation after the program launch. We were thus not able to design pre- and post-study tests to assess quantitative changes in the same program participants. Second, our study recruitment was impacted by the coronavirus pandemic and the PCPs’ availability. The response rate (10%) among the spoke learners was low, so the results should not be interpreted as representing the entire spokes population. The low response rate could be attributed to their limited program engagement. The survey sample size was too small to show statistical significance, so the survey results should be interpreted only as a supplement to the interview results. Third, the Cancer ECHO program recruitment was an ongoing process. Program participants joined the program at different times. Our interviewees and survey respondents did not necessarily enroll in the program at its launch. Their experiences and self-perceived changes are thus limited to their participation.

## Conclusion

This exploratory study showed that the participants and target audience of the Cancer ECHO program in its pilot year appreciated the application of the ECHO model to the subject of cancer control. Its strengths suggest a unique role for the ECHO model among other physician-targetted cancer control interventions. The Cancer ECHO program could be an effective educational means of improving the cancer control capacity of the health care workforce, especially for PCPs. The discrepancy identified between the participants’ and non-participants’ perceived strengths of the program and the relatively low PCP participation rate warrant a further investigation before undertaking a full-scale effectiveness study.

## Supplementary Information

**Additional file 1.** Survey questions.

**Additional file 2.** Survey results on self-reported burnout change.

## Data Availability

The data supporting the findings are contained within the manuscript and its Additional files. The surveys and verbatim analyses of the semi-structured interviews are stored at Indiana University Purdue University Indianapolis. They are available from the corresponding author on reasonable request.
