# Supplementary material for: An evaluation of an Extension for Community Healthcare Outcomes (ECHO) intervention in cancer prevention and survivorship care
Source: BMC Med Inform Decis Mak. 2022 May 17;22:135. doi: 10.1186/s12911-022-01874-x (PMC9112252; doi:10.1186/s12911-022-01874-x)
Supplement: Supplementary file 1 — Additional file 1. Survey questions. [file 12911_2022_1874_MOESM1_ESM.docx]

Additional File 1. Survey Questions

Note: Only related contents items are presented shown here. A complete form of the survey instrument is available from the authors upon request.

Which of the following best describes your role in Cancer ECHO? (If you have an ECHO Record ID, you are enrolled in the Cancer ECHO program)

o Group 1. I did NOT enroll in Cancer ECHO, I was a GUEST in teleECHO clinics (1)

o Group 2. I did NOT enroll in or participate in Cancer ECHO, I ONLY heard about Cancer ECHO (I received Announcement/Recruitment emails, etc) (2)

o Group 3. I have ENROLLED in Cancer ECHO, but I have NEVER participated in any Cancer ECHO clinic session (3)

o Group 4. I am an ENROLLED 'Hub' member, and I have PARTICIPATED in Cancer ECHO clinic sessions (> =1 time) (4)

o Group 5. I am an ENROLLED 'Spoke' member, and I have PARTICIPATED in Cancer ECHO clinic sessions (>= 1 time) (5)

Continued question about your role in Cancer ECHO

o Group 4.1. I am a facilitator in the 'Hub' team (1)

o Group 4.2 I am a subject-matter in the 'Hub' team (2)

o Group 4.3 I am an administrative in the 'Hub' team (Program Director, Coordinator, etc.) (3)

o Group 4.4 I am playing other roles in the 'Hub' team (4)

o Group 5.1 I have PRESENTED a clinical case at a Cancer ECHO clinic (5)

o Group 5.2 I have NOT PRESENTED a clinical case at Cancer ECHO clinic (6)

o Group 5.3 I have attended Cancer ECHO, about 1 time/month or more (> = 50% of the sessions) (7)

o Group 5.4 I have attended Cancer ECHO, less than 1 time/month ( (8)

o (Group 1/2/3.1) I am a wrong target for Cancer ECHO, I do not have a chance to see patients with the needs of cancer prevention or survivorship care (9)

o (Group 1/2/3.2) I have a chance to see patients with the needs of cancer prevention or survivorship care (10)

o (Group 1/2/3.3) I am in the team that sees patients with the needs of cancer prevention or survivorship care, though I do not see them personally (11)

o Other (12)

What is your gender?

o Female (1)

o Male (2)

o Other (3) ________________________________________________

What is the type of your practice?

o Solo practice (1)

o Group practice (2)

o Health maintenance organization (HMO) (4)

o Hospital-based (5)

o Other (3) ________________________________________________

What is the setting of your practice?

o Urban (1)

o Suburban (2)

o Rural (3)

o Other (4) ________________________________________________

Did IU Cancer ECHO sessions meet your expectations?

o Far exceeds expectations

o Exceeds expectations

o Equals expectations

o Short of expectations

o Far short of expectations

Did your KNOWLEDGE of cancer prevention and survivorship care INCREASE, during the period of Cancer ECHO (09/2019 to present)? Of note, the answer should be regardless of your attendance on Cancer teleECHO sessions, the improvement can be from ANY REASON including Cancer ECHO.

o Definitely yes

o Probably yes

o Might or might not

o Probably not

o Definitely not

Did you feel MORE CONFIDENT in managing the complex of cancer prevention and survivorship care during the period of Cancer ECHO (09/2019 to present)? Regardless of your attendance on teleECHO sessions, the improvement can be from ANY REASON, including Cancer ECHO.

o Definitely yes

o Probably yes

o Might or might not

o Probably not

o Definitely not

Did you feel MORE CONFIDENT in managing the complex of cancer prevention and survivorship care during the period of Cancer ECHO (09/2019 to present)? Regardless of your attendance on teleECHO sessions, the improvement can be from ANY REASON, including Cancer ECHO.

o Definitely yes

o Probably yes

o Might or might not

o Probably not

o Definitely not

Did you feel MORE CONFIDENT in managing the complex of cancer prevention and survivorship care during the period of Cancer ECHO (09/2019 to present)? Regardless of your attendance on teleECHO sessions, the improvement can be from ANY REASON, including Cancer ECHO.

o Definitely yes

o Probably yes

o Might or might not

o Probably not

o Definitely not

Did your PROFESSIONAL PRACTICE of managing cancer prevention and survivorship care IMPROVE during the period of Cancer ECHO (09/2019 to present)? Regardless of your attendance on teleECHO sessions, the improvement can be from ANY REASON, including Cancer ECHO.

o Definitely yes

o Probably yes

o Might or might not

o Probably not

o Definitely not

I feel more EMOTIONALLY EXHAUSTED at work compared to 09/2019 (when Cancer ECHO launched). Regardless of your attendance of teleECHO sessions, the worsening can be due to ANY REASON, including Cancer ECHO.

o Strongly agree

o Agree

o Somewhat agree

o Neither agree nor disagree

o Somewhat disagree

o Disagree

o Strongly disagree

Cancer ECHO has improved my emotional exhaustion at work.

o Strongly agree

o Agree

o Somewhat agree

o Neither agree nor disagree

o Somewhat disagree

o Disagree

o Strongly disagree

I feel less sensitive to my patients' feelings now compared to 09/2019 (when Cancer ECHO launched). Regardless of your attendance of teleECHO sessions, the worsening can be due to ANY REASON, including Cancer ECHO.

o Strongly agree

o Agree

o Somewhat agree

o Neither agree nor disagree

o Somewhat disagree

o Disagree

o Strongly disagree

Cancer ECHO has improved my sensitivity to my patients' feelings

o Strongly agree

o Agree

o Somewhat agree

o Neither agree nor disagree

o Somewhat disagree

o Disagree

o Strongly disagree
