# Supplementary figures and images for: An evaluation of an Extension for Community Healthcare Outcomes (ECHO) intervention in cancer prevention and survivorship care
Source: BMC Med Inform Decis Mak. 2022 May 17;22:135. doi: 10.1186/s12911-022-01874-x (PMC9112252; doi:10.1186/s12911-022-01874-x)

Additional File 2. Survey Results on Self-Reported Burnout Change


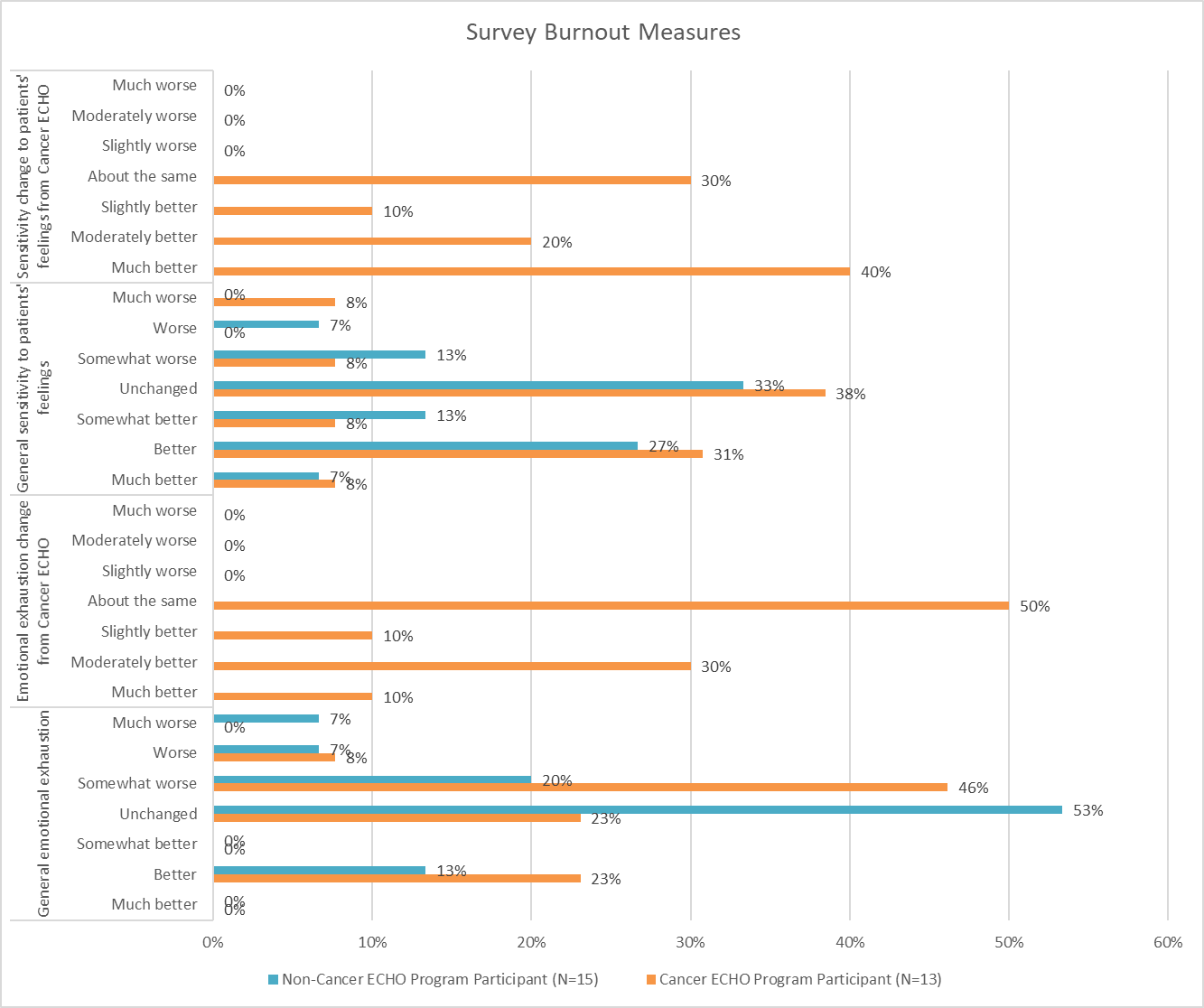

Supplement: Supplementary file 2 — Additional file 2. Survey results on self-reported burnout change. [file 12911_2022_1874_MOESM2_ESM.docx]
